# Promising Perspectives for Detection, Identification, and Quantification of Plant Pathogenic Fungi and Oomycetes through Targeting Mitochondrial DNA

**DOI:** 10.3390/ijms21072645

**Published:** 2020-04-10

**Authors:** Tomasz Kulik, Katarzyna Bilska, Maciej Żelechowski

**Affiliations:** Department of Botany and Nature Protection, University of Warmia and Mazury in Olsztyn, Plac Łódzki 1, 10-727 Olsztyn, Poland; katarzyna.bilska@uwm.edu.pl (K.B.);

**Keywords:** plant pathogenic fungi and oomycetes, detection, identification, quantification, DNA-based markers, mitochondrial DNA

## Abstract

Fungi and oomycetes encompass many pathogens affecting crops worldwide. Their effective control requires screening pathogens across the local and international trade networks along with the monitoring of pathogen inocula in the field. Fundamentals to all of these concerns are their efficient detection, identification, and quantification. The use of molecular markers showed the best promise in the field of plant pathogen diagnostics. However, despite the unquestionable benefits of DNA-based methods, two significant limitations are associated with their use. The first limitation concerns the insufficient level of sensitivity due to the very low and uneven distribution of pathogens in plant material. The second limitation pertains to the inability of widely used diagnostic assays to detect cryptic species. Targeting mtDNA appears to provide a solution to these challenges. Its high copy number in microbial cells makes mtDNA an attractive target for developing highly sensitive assays. In addition, previous studies on different pathogen taxa indicated that mitogenome sequence variation could improve cryptic species delimitation accuracy. This review sheds light on the potential application of mtDNA for pathogen diagnostics. This paper covers a brief description of qPCR and DNA barcoding as two major strategies enabling the diagnostics of plant pathogenic fungi and oomycetes. Both strategies are discussed along with the potential use of mtDNA, including their strengths and weaknesses.

## 1. Introduction

Fungi and oomycetes include a large number of pathogens having an enormous impact on the production and quality of food, fiber and biofuel crops worldwide [1,2,3,4]. Both groups of microorganisms belong to different phyla but are morphologically similar, exhibit filamentous growth and an osmotrophic lifestyle [5,6].

From about 100,000 described fungal species, around 20% comprise plant pathogens responsible for 70–80% of plant diseases [7,8]. Among the top ten fungal species/complexes of worldwide scientific and economic importance [9], seven: *Magnaporthe oryzae*, *Puccinia* spp., *Fusarium graminearum* sensu stricto (s.s.), *Blumeria graminis*, *Zymoseptoria tritici* (formerly *Mycosphaerella graminicola*), *Ustilago maydis*, and *Melampsora lini* are associated with cereals. A high priority cereal pathogen can also encompass *Rhizoctonia solani* causing sheath blight and yield reduction in rice and other crops [9].

Their importance is underscored by the fact that more than one-half of the world’s daily caloric intake is derived directly from cereal grain consumption [10]. Other highly destructive plant pathogens include *Botrytis cinerea*, the causal agent of gray mold on a wide range of fruit and vegetables [11], *Fusarium oxysporum* species complex (FOSC), responsible for vascular wilt diseases of economically important crops [12], *Colletotrichum* spp., causing anthracnose on more than 30 plant genera [13] and *Phakopsora pachyrhizi*, the causal agent of Asian soybean rust [9].

Besides yield reductions, many plant pathogenic as well as spoilage fungi create a considerable qualitative loss of food and feed by contamination of plant material with mycotoxins [14,15]. The genera of mycotoxigenic fungi are mainly represented by *Aspergillus*, *Penicillium,* and *Fusarium* [16]. Mycotoxins produced by these fungi have well-documented negative effects on humans and animals [17]. 

Oomycetes are the second major group of eukaryotic pathogens causing plant diseases. The most economically important pathogens include *Phytophthora* species, such as the potato late blight *Phytophthora infestans* [3,18,19], *Phytophthora sojae* which rots the roots of soybeans, *Phytophthora capsici* a devastating pathogen of vegetable crops, and *P. cinnamomi* attacking a wide range of plant species around the world including both oaks and chestnuts [20]. Other destructive species include *P. palmivora*, causing cocoa black pod, and *P. ramorum*, causing extensive damage and death to trees [3,21]. Moreover, economically important oomycete pathogens include members of the genus *Pythium* and downy mildews [3,22,23]. 

Unlike typical saprotrophic microorganisms, many plant pathogenic species appear not to be driven by dispersal limitation or climate [24,25]. Plant pathogens can spread by a variety of means. In fungi, both asexual and sexual spores can disperse in large quantities by airflow and raindrops releasing millions of tons of spores into the atmosphere every year [26]. However, recently, the increase in foodstuff trade has been recognized as the most significant risk factor contributing to the introduction of invasive plant pathogens into new locations [1,27,28]. Therefore, the control of plant pathogens requires biosecurity and quarantine systems in international trade to minimize their spread [4]. The eight fungal species/complexes of the highest importance [9] are: *Magnaporthe oryzae*, *Botrytis cinerea*, *Fusarium graminearum* s.s., *Fusarium oxysporum* species complex, *Zymoseptoria tritici*, *Colletotrichum* species complex, *Melampsora lini,* and *Rhizoctonia solani* that can spread through transported plant material [25,29,30,31,32]. 

To date, however, the most striking negative consequences of the spread of invasive pathogens can be derived from the introduction of plant pathogenic oomycetes which have caused damage to crops, ornamental plants, and forests on a global scale for centuries [33]. The most destructive oomycetes *Phytophthora infestans*, *Phytophthora palmivora*, *Phytophthora ramorum*, and *Plasmopara obducens* together with their pathology, importance and impact are extensively discussed in Brasier (2008) [33] and Derevnina et al. (2016) [3]. It is now generally accepted that their success in invading new geographic areas results from their potential to spread through the transport of infected or infested plant material or water, a flexible mating system, and their ability to adapt rapidly to new hosts and environments [3].

Besides international biosecurity regulatory mechanisms, the monitoring of the movement of pathogens across the plant trade network is crucial to reducing the spread of invasive pathogens [32]. In addition, the monitoring of pathogen inocula in the field is an important approach to provide continuously updated data on microbial surveillance and adaptation [1,34]. 

A fundamental aspect to all of these concerns is the efficient identification of pathogens. Effective detection of pathogens provides all necessary data to undertake more predictive and preventative actions [4]. Such knowledge includes information on species, cryptic species, races, formae speciales, anastomosis groups, and mating types. However, the efficient detection of plant pathogens is still a challenging task. Microbial biomass may persist at relatively low levels as mycelium colonizing the tissue internally and/or spores infesting the surface of a given material, which cannot be often detectable by visual means. Notably, the methods applied to detect pathogen diversity should be of high sensitivity, reliability, and rapidity. In addition, today, it has become imperative to effectively detect morphologically indistinguishable cryptic species within fungal complexes [35,36]. 

Among the number of diagnostic methods for fungi and oomycetes, molecular identification (DNA-based) showed the best promise in the field of plant pathogen diagnostics. However, despite the unquestionable benefits of DNA-based methods, two significant limitations are associated with its use. The first limitation concerns the insufficient level of sensitivity of diagnostic assays, which is mainly associated with targeting single-copy nuclear genes. The second major limitation pertains to the inability of widely used diagnostic assays to detect cryptic species. 

This review aims to provide the potential application of mitochondrial DNA (mtDNA) in the field of eukaryotic plant pathogen detection. Our paper covers brief descriptions of two major strategies enabling both quantification and identification of plant pathogenic fungi and oomycetes. The first one involves qPCR technology for pathogen detection/quantification, and the second one describes DNA barcoding for identification of fungi and oomycetes to the species level. Both strategies are discussed along with the potential use of mtDNA including their strengths and weaknesses.

## 2. A Brief Overview of qPCR for the Detection and Quantification of Plant Pathogenic Fungi and Oomycetes

The taxonomy provides a basic understanding of fungal biodiversity which is necessary for effective plant disease management. Undoubtedly, taxonomic information is essential in communicating between scientists, the regulatory authorities and related organizations [37,38]. Traditional taxonomic research allows for the identification of morphologically different fungal species. However, time-consuming and laborious culture-based methods have frequently been criticized by mycologists mostly due to difficulties in the culturing of some species, and the inability to quantify the pathogens [39,40,41].

Molecular approaches have led to greater confidence and accuracy in the identification of plant pathogenic fungi and oomycetes [39,42,43]. The most prevalent molecular method relies upon polymerase chain reaction (PCR), which has long been used in the field of plant pathology [44,45,46,47]. The major advantage of PCR over older traditional methods is the abortion of laborious and time-consuming culturing of microorganisms [39,47]. Microbial templates used for PCR may be extracted from diseased plant material or samples from contaminated habitats such as soil, water, or air [47,48,49,50]. The high reliability of PCR is mainly achieved by the incorporation of specific primers, which prevent undesired amplification [51]. By choosing appropriate primers, it is possible to target different species [52,53], races [54,55], formae speciales [56,57,58,59], and genotypes [60,61,62]. Many different PCR-based assays for diagnosis and monitoring of plant pathogens have been extensively reviewed in Singh and Gupta (2017) [63].

The other remarkable advantage of PCR is its high sensitivity. Even very low concentrations of pathogen DNA present in a complex DNA mixture could be effectively amplified and visualized. The major limitation of conventional PCR results from the requirement of time-consuming gel electrophoresis with visualization, which is not optimal for the detection of trace quantities of DNA due to its lower sensitivity [64]. In addition, the high risk of carry-over amplicons from previous amplification has been the main impediment to using PCR routinely in diagnostic laboratories [65,66,67]. More importantly, like other minor molecular techniques such as loop-mediated isothermal amplification (LAMP) [68] and dot blot hybridization [69], conventional PCR does not provide quantitative data [70,71].

When it comes to quantification of microbes, real-time PCR (syn. qPCR (quantitative PCR) is a major breakthrough revolutionizing plant pathology for more than two decades. Real-time PCR enables the monitoring of fluorescent signal which is proportional to the amount of amplicon produced in each cycle and can be generated by an intercalating dye or from the breakdown of a dye-labeled probe during amplification [72,73]. This allows the detection and quantification of specific DNA molecules either for their presence or absence or for their amount [74].

The major advantages of real-time PCR are: ease-of-use, closed-tube format, rapidity, simplicity to perform analysis, and the extremely wide dynamic range of quantification (more than eight orders of magnitude) [75]. Notably, compared to endpoint PCR, real-time PCR technology is more sensitive due to a fluorescent marker [76,77]. In addition, the use of hydrolysis probes (TaqMan) offers an additional level of specificity [77]. 

Most qPCR-based approaches for plant pathogens have been designed to detect and quantify traditionally defined species (Table 1 and Table 2). 

Among nuclear targets, ribosomal DNA (rDNA) containing protein-coding genes, introns, and intergenic spacers showed the best promise for plant pathogen quantification. The major advantages of targeting rDNA are: (i) simplicity to obtain the PCR products for sequencing due to established universal fungal primers, (ii) multi-copy nature offering increased amplification success of rDNA over single-copy targets, and (iii) additional ability to perform identification of species through sequence similarity searches against the large number of rDNA sequences in GenBank. Thus, it is not surprising that among 20 qPCR approaches for the most economically important plant pathogenic fungi, nearly half have been designed based on rDNA sequences (Table 1). 

rDNA appears to have a higher resolving capacity for discriminating plant pathogenic oomycetes. Most of the diagnostic assays designed to detect and quantify this group of plant pathogens were developed based on rDNA. 

Recently, many traditionally defined plant pathogenic fungal species have been found to be species complexes containing a large number of cryptic (syn. phylogenetic) species [113,114,115,116,117]. The most important examples of such economically significant complexes are: *Colletotrichum acutatum*, *Colletotrichum gloeosporioides*, *F. graminearum*, *F. oxysporum,* and *Rhizoctonia solani*. Deciphering cryptic species diversity appears to be critical for scientists to determine their geographical distribution and host range, to identify their biosafety and biosecurity threads, and to understand the evolution of pathogenicity [4,113,118]. Implementation of real-time technology for the diagnostics of phylogenetic species remains a considerable challenge mostly due to: (i) the need for initial screening of a large set of candidate genes to reveal a sufficient level of sequence polymorphism among closely related species and (ii) the continuous changes in fungal taxonomy which still undergo significant progress. Therefore, to date, only three different qPCR approaches have been designed to detect cryptic species among fungal plant pathogens (Table 2).

Ideal DNA targets for such assays cannot have non-orthologous copies. Thus, it appears that single-copy protein genes provide robust and reliable targets for successful detection of cryptic species within fungal complexes [119]. We hypothesize that the rapidly increasing progress in the sequencing of fungal genomes will accelerate the development of new quantitative assays through recognition of yet undescribed species and rapid detection of sequence polymorphisms to enable their quick differentiation. 

Besides species determination, qPCR technology could be successfully used to study the genetic structures of field populations at the intraspecific level. For this purpose, numerous qPCR approaches have been designed to obtain additional genetic data regarding formae speciales [107,109], mating types [120,121], and mycotoxin genotypes [60,122]. 

Highly sensitive detection of pathogens is increasingly important. Pathogens may survive in environmental material or host DNA in relatively low quantities making their efficient detection challenging to analysts [83,123]. Microbial detection by any molecular method requires the use of extraction procedure that efficiently lyse cells and recovers DNA suitable for amplification. In general, difficulties in the detection of plant pathogens through PCR may be linked to either insufficient recovery of pathogen DNA for amplification or reduced limit of detection (LOD) of a given assay. Amongst plant pathogens, extraction of fungal DNA remains the most problematic mostly due to: (i) chitinous cell walls making cell lysis and DNA extraction inefficient [124], (ii) hyphal and patchy penetration of substrates (hyphae is difficult to concentrate by centrifugation) [125], and (iii) fungal spores can serve as an additional source of a DNA template for qPCR, however, some fungal genera such as *Alternaria* or *Fusarium* display reduced sporulation in relation to the growth of mycelia [126]. 

As shown in Table 1 and Table 2, individual qPCR approaches designed to detect plant pathogenic fungi and oomycetes may largely differ in their detection limits. High differences can be found when comparing qPCR approaches for the same species and even targeting the same locus. A comparison of methodologies from various laboratories used to establish LOD values may provide some clues about these differences. First, the authors appear to use different cut-off values below which quantification cycle values (Cq) indicate positive results [127]. This strategy allows to reduce the risk of false-positive results which may come from background noise. Secondly, it appears that LOD values are established using various criteria. Some authors establish LOD based on serial dilutions of genomic DNA extracted from pure pathogen biomass, while others define LOD as the lowest amount of genomic DNA that could be detectable from infected plant material or substrate. As shown by Martin et al. (2004) [128], LOD can significantly reduce when spiking target DNA with background DNA. 

Finally, it appears that LOD values may also be affected by the type of real-time PCR format used to monitor the amplification process. For example, the widely used SybrGreen format has been found to be more sensitive than TaqMan [79], although the first one lacks consistently reproducible quantification when the target DNA is present in low quantities [129,130]. Samples exhibiting low pathogen load produce weak fluorescence signals resulting in high (or late) quantification cycle values (Cq). Detecting low copy template DNA is often error-prone mostly due to lower reproducibility of the results and the risk of late false-positive results generated by background noise [127]. To prevent it, the use of assays targeting repeated genomic sequences rather than the single copy gene assays is recommended [131,132]. The most commonly used methods utilizing multi-copy sequences are rDNA-based approaches. Dramatic differences in the detection limits of both rDNA and single-copy based assay were previously demonstrated by Suarez et al. (2005) [79]. Using the same biological samples, it was found that IGS-based (intergenic spacer) assay improved the detection limit of *Botrytis cinerea* by 1000-fold, as compared with single-copy cutinase-A assay [82]. 

However, despite the above benefit of utilizing rDNA, other significant diagnostic limitations are associated with its use. Some morphologically defined plant pathogenic fungi (e.g., *Fusarium* species) cannot be discriminated based on rDNA. In addition, the reduced discriminating power of rDNA limits recognition of morphologically indistinguishable cryptic species within the most economically important species complexes of plant pathogenic fungi.

## 3. A Brief Overview of DNA Barcoding for Identification of Plant Pathogenic Fungi and Oomycetes 

DNA barcoding is a relatively new method for identification of any species, which is now being applied to taxa across the tree of life, including fungi and oomycetes [133,134,135,136,137,138]. DNA barcode may be defined as a short, unique DNA sequence pattern (ca. 400–800 bp) which, in theory, can be quickly amplified, sequenced, and characterized by specific software to identify species [4,139]. In 2003, the DNA barcoding initiative began, searching to find a universal barcode for taxon identification [140]. The first proposed barcode was a mitochondrial cytochrome c oxidase subunit 1 (*cox1*, syn. *co1*) [140], tagging taxons in the animal kingdom. However, for barcoding of fungi, *cox1* was excluded, mainly due to insufficient variation and mosaic distribution of introns, which bias amplification efficiency [136,141,142]. For the barcoding of fungi and oomycetes, the internal transcribed spacer (ITS) region has been proposed [141,143,144]. ITS is localized between the small-subunit ribosomal RNA (rRNA) and large-subunit rRNA genes in the chromosome. In eukaryotes, those genes occur in tandem repeats with thousands of copies, separated by regions of non-transcribed DNA named intergenic spacer (IGS) or non-transcribed spacer (NTS). ITS region appears to have the highest probability of reliable identification for fungi, with the most clearly defined barcode gap between inter- and intraspecific variation [143]. However, its broad utility as a species marker for fungi has been criticized, because: (i) divergent intragenomic ITS sequences are found in several fungal groups [145] and (ii) the low-resolution power in discriminating closely related species, including cryptic species [141,145]. Many plant pathogenic fungi, e.g., *Alternaria, Botryosphaeria, Cercospora, Diaporthe,* and *Fusarium* cannot be identified to the species level based on the ITS sequence [141,146,147]. In addition, in the downy mildew genera *Basidiophora*, *Plasmopara*, *Plasmoverna,* and relatives, the ITS region contains large insertions (often longer than 2 kb) raising considerable difficulties in amplification and subsequent sequencing [148].

The ITS region also exhibits limitations in the discrimination of the species of oomycetes. In *Phytophthora* spp. [149,150,151] and *Peronospora* spp. [152,153], ITS regions show insufficient intraspecies variability for reliable determination of some species. In other economically important genera such as *Bremia* and *Plasmopara* of the Peronosporales, ITS regions contain long repetitive insertions, which largely limit amplification success of this barcode [148,152].

In fungi, other nuclear, single-copy protein-coding genes have been extensively tested for their efficacy in discrimination of plant pathogenic species. The most widely used genes include: the largest (*rpb*1) and the second-largest (*rpb2*) subunits of RNA polymerase [154], translation elongation factor 1-alpha (*tef1*) [155,156,157,158], β-tubulin [159,160,161], the mini-chromosome maintenance protein (*mcm7*) [162], calmodulin (*CaM*) [163], and topoisomerase I (*top1*) gene [164,165]. Notably, sequence polymorphisms of protein-coding genes offer an improved species resolution than ITS, although low amplification success often excludes them as candidates for routine barcoding of fungi [143]. 

It is worth noting, however, that reliable identification of cryptic species, which diverged relatively recently, requires more than one molecular marker. Thus, recognition of these masked species relies heavily on Genealogical Concordance Phylogenetic Species (GCPS), which involves multi-gene phylogenetic analyses [166,167,168]. Although GCPS seems to have profound implications for fungal control and quarantine [35], the requirement for multi-locus sequencing coupled to bioinformatic and phylogenetic analyses makes this method time-consuming and expertise specific. In addition, shortcomings related to decreased sensitivity of housekeeping genes largely limits the adoption of this method for environmental applications. 

## 4. Mitogenome Characteristics of Fungi and Oomycetes

Most fungal lineages harbor mitochondria, the energy factories in eukaryotic cells. Besides energy production, mitochondria contribute to various cellular and organism functions [169,170,171,172] and can be involved in antifungal resistance, virulence and pathogenicity [172,173]. It is therefore not surprising that special attention has been drawn to study mitochondrial structure and function and its own genome (mitogenome) [170,174]. 

Nowadays, the relatively small size of fungal mitogenomes allows their robust study as an entity, especially with massively parallel sequencing platforms [175,176]. Currently, 653 fungal mitogenomes are available in the GenBank database with a large portion representing plant pathogenic species. Fungal mitogenomes vary widely in size, structure and in the content and order of their genes [174,175,177]. Most of them tend to be AT-rich and can exist in either linear or circular form [174]. Fungal mitogenomes range from 19 to over 200 kbs [178,179], however fungi in the Neocallimastigales order have completely lost their mitochondrial genome [174]. Fungi can exhibit a diverse inheritance models: uniparental, biparental, and a mixture of both [180,181,182,183]. 

In general, fungal mitogenomes usually contain 14 protein-encoded genes: (i) *atp6*, *atp8*, *atp9* (encoding subunits of ATP synthase), *cob* (*encoding* cytochrome b), *cox1-3* (encoding cytochrome oxidase subunits), *nad1-6*, and *nad4L* (encoding the NADH dehydrogenase subunits), which are all implicated in oxidative phosphorylation and the production of ATP; (ii) two genes of rRNA (*rns* and *rnl*) and a gene (*rps3*) are responsible for the translation and the composition of the small and large subunits of the mitochondrial ribosome; and (iii) a set of genes (*trn*) for tRNAs (ranging from 8–24) [174,177]. They also contain a variable number of self-splicing introns which are partial ribozymes. Group I introns typically found in fungi harbor homing endonucleases (HEGs) with LAGLIDADG and GIY-YIG motifs, which can promote their mobility among different lineages. Less common in fungi, group II introns usually contain reverse transcriptases. Homing endonucleases and transcriptases are selfish because they pose no obvious value to their host genomes [174,184,185,186,187]. 

Fungi exhibit remarkable variation in intron content [187], which can also be observed in plant pathogens. The *Fusarium proliferatum* mitogenome, for example, contains only a single intron [188], which is in contrast to *R. solani* which contains dozens of introns [179]. In addition, at the population or even strain level, these fungi can differ in intron content [189,190]. However, the same introns may also be found in evolutionarily distant lineages and their widespread distribution in fungi may be due to the intron-rich progenitor from which extensive intron loss has occurred [187,189]. In addition to intron loss, the infective nature of HEGs is believed to cause the observed variation in the intron content between different lineages. It should also be noted that exploration of the history of introns and associated HEGs across organisms is rather challenging for researchers, mostly because of the difficulty in detecting their gain and loss events in fungi [189,191]. 

Currently, only 20 oomycete mitogenomes are available in the GenBank database with a significant portion representing plant pathogenic species. In this group of plant pathogens, both size and genetic structure of mitogenomes have been proven to be highly variable, from approximately 37 kb for *Phytophthora infestans* to the largest around 60 kb mitogenomes of *Pythium* spp. In contrast to fungi, mitogenomes of oomycetes lack introns, and the large inverted repeats which accumulate are the largest contributor to mitogenome size variation, especially in mitogenomes of *Pythium* spp. Yuan et al. (2017) [192] showed that the ancestral expansion of inverted repeats resulted in gene duplication in the Pythiales and Saprolegniales compared with the Peronosporales. It has been indicated that whole mitogenome analysis appears to provide a view of the evolutionary history and phylogeography of the oomycetes and a comparison of sequence data from herbaria and living organisms can detect evolutionary events leading to the divergence of this important group of pathogens [192,193].

## 5. Targeting mtDNA Improves Detection and Quantification of Plant Pathogenic Fungi and Oomycetes

The demand for highly sensitive detection of pathogens has become an important issue in recent years [46,194]. Targeting mtDNA seems an ideal solution, mainly due to the multi-copy nature of mtDNA in eukaryotic cells [174,195,196]. 

Mitochondrial-based qPCR (mtqPCR) methods were developed and used to detect *Aspergillus fumigatus* in serum [197], bronchoalveolar lavage fluids, and tissue biopsy specimens [198]. The analytical sensitivity of the assay was one *A. fumigatus* conidium per reaction [198]. It has been demonstrated that mt-based detection of *A. fumigatus* in patients with risk factors for invasive aspergillosis showed lower LOD than other multi-copy rDNA assays [199]. 

For plant pathogens, the first mtqPCR approach was developed by Gao et al. (2004) [200] for both absolute and relative quantification of *Fusarium virguliforme* (formerly *Fusarium* *solani f*. *sp*. *glycines*) causing sudden death syndrome (SDS), a widespread and destructive soybean disease. The pathogen biomass was quantified utilizing the mitochondrial small subunit (mtSSU) rDNA region. The fungus was detected in soybean plants using SybrGreen format for contents as low as 9.0 × 10^–5^ ng in the absolute qPCR assays. 

In another study, Li et al. (2008) [201] developed a TaqMan assay for the detection of *F. virguliforme* from soybean roots. A specific minor-groove binding (MGB) probe and primer set were derived from the sequences of the mtSSU. Unfortunately, the authors of the above study did not evaluate LOD and/or limit of quantification (LOQ) of the developed assay. A more recent study by Mbofung et al. (2011) [202] showed, however, that mtqPCR assays for *F. virguliforme* give positive results from the other SDS-causing Fusaria, as well as DNA from some *F. solani* strains. It is worth noting that these assays were developed prior to recent findings showing that the mtSSU locus is unable to resolve species boundaries within the SDS-bean root rot (BRR) clade of the *F. solani* species complex due to the conserved nature of this locus [202,203,204]. Unfortunately, to date, the GenBank database lacks complete mitogenome sequences from *F. solani* species complex, making it impossible to investigate which mitochondrial locus could be useful for reliable discrimination between various members of this complex.

Specific quantification of the cryptic species *Fusarium graminearum* s.s. using the mtqPCR approach has been recently demonstrated by Kulik et al. (2015) [53]. This ascomycete fungus is the major cause of Fusarium head blight (FHB), a devastating disease of small grain cereals worldwide. *F. graminearum* s.s. belongs to the monophyletic fungal complex referred to as *F. graminearum* species complex (FGSC) encompassing 16 cryptic species [205]. Primers and a MGB probe were designed utilizing sequence polymorphism within the intronic sequence of *cob* gene. The LOQ of the FgMito assay (0.2 pg) is the equivalent of approximately five haploid cells of *F. graminearum* s.s, while the LOD of the assay was determined between 0.2 and 0.06 pg. The mean of these two concentrations equals approximately three haploid fungal cells. Comparison of FgMito assay with the assay targeting the nuclear genome [105] on naturally contaminated grains showed increased sensitivity of a mitochondrial-based approach in quantifying *F. graminearum* s.s. 

In a more recent study, Bilska et al. (2018) [83] designed the mtqPCR approach for quantification of *F. culmorum*, a ubiquitous, soil-borne fungus causing foot and root rot and Fusarium head blight on cereals. Primers and MGB probe were designed based on the intronic sequence within the *cox2* gene. The LOQ of the FcMito assay was determined as 0.05 pg. This is 18-fold lower than LOQ of another *F. culmorum* specific TaqMan assay targeting the nuclear genome [85]. The LOD of the new mitochondrial assay was determined between 0.05 and 0.005 pg, which corresponds to less than one and a quarter of the haploid cell of *F. culmorum*. This study also found a positive correlation between the *F. culmorum* mtDNA and the total trichothecenes present in naturally contaminated grains.

The use of mitochondrial DNA for quantification of oomycetes is more frequent than in fungi. It is worth noting that, in contrast to fungi, mitogenomes of oomycetes do not contain introns—making their prior amplification for sequencing and primer design easier. 

One of the first mt-based assays for plant pathogenic oomycetes were developed for species that damage forests and trees. Tooley et al. (2006) [95] designed the primers and probes to quantify both pathogen (*P. ramorum*) and plant DNA in multiplex reactions enabling minimized underestimation of quantity and false negatives via PCR inhibition. The limit of detection of *P. ramorum* DNA was 1 fg of genomic DNA, much lower than for many other described PCR procedures for detecting *Phytophthora species*. 

In another study, Martin et al. (2004) [128] designed the genus and species-specific markers targeting *cox1* and *cox2* genes. Using DNA from purified cultures, the *Phytophthora* genus-specific primer pair successfully produced amplicon from all 45 *Phytophthora* spp. tested. Using purified pathogen DNA, the limit of detection for *P. ramorum* using this marker system was approximately 2.0 fg of total DNA.

Bilodeau et al. (2014) [206] developed mitochondrial markers utilizing *atp9* gene, intergenic spacer sequences and three tRNAs (*trnM*-*trnP*-*trnM*) to detect and quantify *Phytophthora* species. TaqMan markers encompassed genus-specific and species-specific assays for 13 species and the *P. citricola* species complex. The markers developed by Bilodeau et al. (2014) [206] were validated against a range of oomycetes from various geographic origins including: *Phytophthora* spp., *Pythium* spp., *Phytopythium* sp., as well as different plant DNA. Importantly, in silico analysis showed that species-specific assays could be developed for at least 70 other plant pathogenic oomycetes. 

A more recent study by Yuan et al. (2017) [192] showed that among mitochondrial genes *rpl6*, *rps10*, *atp8*, *nad11*, *rps11*, *rps2*, *rps3*, *nad9*, and *rps4* show similar resolution as rDNA and could be used for the identification of species in the Peronosporales. It has been suggested that the high sensitivity of mitochondrial markers can allow establishing more precise monitoring and control strategies for this group of important pathogens [192].

## 6. Perspectives of Targeting mtDNA for Barcoding of Plant Pathogenic Fungi and Oomycetes

In one of the first studies, Seifert et al. (2007) [142] demonstrated that a mitochondrial *cox1* gene could be highly effective in the resolution of *Penicillium* species. The authors of the above paper designed primers that allowed generating amplicons from multiple strains encompassing 58 species of *Penicillium* subgenus *Penicillium* and 12 allied species. The majority of the analyzed species have been resolved based on *cox1* sequence data and the amplification of *cox1* proved to be more efficient than the other nuclear genes.

Disappointing results on the use of *cox1* for identification of plant pathogenic Fusaria have been provided by Gilmore et al. (2009) [207]. The major limitations which excluded *cox1* as a barcode for tagging *Fusarium* species were: (i) multiple copies (paralogues), (ii) a lack of a species-level resolution within homologous copies, (iii) and the presence of introns affecting amplification efficiency. To date, however, the suitability of other mt-genes for identification of Fusaria has not been tested, due in large part to a lack of available mitogenome sequences. 

As mentioned earlier, the presence of mobile introns in fungal mitogenomes appears to be the major source of difficulties in analyzing mitogenome data through PCR and sequencing [186,208]. Santamaria et al. (2009) [208] proved the irregular distribution of mobile introns [209] in almost all the mitochondrial genes of Ascomycota and revealed that only a few *nad* genes and two rRNA genes do not contain introns, which highlighted their potential applicability for barcoding purposes [208]. 

Further, an in silico study of Vialle et al. (2009) [210] evaluated the potential of 14 mitochondrial genes for barcoding Basidiomycota species. Mitochondrial genes exhibited high discrimination power, highlighting their promising contribution for the resolution of lower-level relationships, however, the revealed intron presence-absence polymorphism within *cox1* and in six other genes, excluded their potential usefulness for tagging members of Basidiomycota. Three genes: *atp6, co3,* and *nad6* have been shown to exhibit promising characteristics for DNA barcoding of Basidiomycetes, however, no single mt-gene gave a better taxonomic resolution than the ITS region [210].

The most recent study by Liang et al. (2017) [211] evaluated the efficacy of mitogenome sequence in the delimitation of four taxonomically challenging cryptic species within *Colletotrichum* *gloeosporioides* sensu lato (*C*. *gloeosporioides*, *C. fructicola*, *C. aenigma*, and *C. siamense* s.l.). Reliable delimitation of phylogenetic species in *C. gloeosporioides* s.l. using prevailing nuclear markers is very challenging due to nascent lineage boundaries. Phylogenetic analysis using mtDNA allowed the generation of a high-resolution phylogeny, recognizing all members of *C. gloeosporioides* s.l. complex. A 142 bp region in the *cox3* ORF was identified showing strong lineage-specific divergence. Interestingly, the authors suggested that intron presence–absence polymorphism contain a phylogenetic signal, which could be used for designing species–specific approaches. Introns located within *cob* and *cox1* genes have been shown to be conserved among all *C. gloeosporioides* s.l. mitogenomes, however, none of them were found in mitogenomes outside of the *C. gloeosporioides* s.l. complex, highlighting their use for potential diagnostic purposes [211]. A species-specific pattern of intron distribution has been also observed in *F. oxysporum* [188] and *Fusarium fujikuroi* species complex (FFSC) [212]. Recently, Gomes et al. (2018) [213] suggested that group I introns are promising targets for developing novel tools for fungicide susceptibility. Some fungicides inhibit self-splicing of group I introns, which is indispensable for pathogen survival under parasitic conditions [213]. Intron distribution has been suggested as an important contributor to the virulence and drug tolerance of human fungal pathogens *Cryptococcus neoformans* and *Cryptococcus gattii*. Unfortunately, our knowledge of the role of introns in antifungal tolerance and virulence of plant pathogenic fungi is still lacking. 

The barcoding of oomycetes based on *cox1* gene has been first demonstrated by Robideau et al. (2011) [144], through sequence comparison from strains representing 23 genera in this important group of pathogens. It has been proven that in some cases cox1 displayed even higher resolution power than ITS. Robideau et al. (2011) [144] suggested that reliable identification of *Pythium* and *Phytophthora* species could be achieved through the combined use of both ITS and *cox1*. However, the amplification success of *cox1* appears to be lineage dependent [214]. To overcome this limitation Choi et al. (2015) [214] proposed incorporation of *cox2* for the barcoding of oomycetes. Primers designed to amplify a portion of *cox2* showed higher amplification success than *cox1*. Remarkably, *cox2* barcode displayed a barcoding gap with relatively higher scores for identification to the species level from both living samples and from historic herbarium specimens [214].

## 7. Conclusions and Future Perspectives

Recent advances in next-generation sequencing have gained a considerable number of openly available microbial mitogenomes. Massively parallel sequencing platforms can produce millions of short sequences in a single run [215], thereby enabling for sequencing of the whole mitogenomes and proving valuable for searching candidate barcodes for pathogen identification [136]. Universal mitochondrial barcode for both fungi and oomycetes does not exist because the discriminatory power of different barcode regions is not uniformly distributed across the lineages [136,141,207,214,216]. For this reason, reliable barcoding of both fungi and oomycetes through mtDNA will require amplification and analysis of multiple barcodes. In the case of fungi, the distribution of some introns and associated HEGs appears to be species-specific, which opens a new window for investigating intronic sequences for diagnostic purposes. This can be only assessed over a significant sample of genera throughout the distributional range.

Despite the unquestionable benefits of NGS (Next Generation Sequencing) applications, sequence length limitations of technologies developed by Illumina, Life Technologies, and Roche justifies the use of short (<500 bp) amplicons for further analyses. Species-level determinations are often impossible for short reads. Enrichment of large fragments of mtDNA especially from environmental samples with long-range PCR could be a viable solution [217,218]. Notably, long-range PCR avoids the risk associated with amplification of NUMTs (nuclear-encoded mitochondrial pseudogenes) and the rearrangements of targeted priming sites with altered gene order [218,219], making sequence assembling less problematic. However, successful amplification with long-range PCR requires high-quality and high-molecular-weight DNA [218], whose recovery from food or environmental samples might pose a considerable challenge. For difficult samples with relatively low-quality DNA, real-time PCR probably remains the best option. Regardless of the selected method, the strong evidence revealed for species-specific polymorphism in fungal mitogenomes opens entirely new directions in fungal diagnostics, which may provide significant benefits in agriculture and food security when the highly sensitive detection of fungi is required.

## Figures and Tables

**Table 1:** Examples of species-specific quantitative polymerase chain reaction (qPCR) approaches for morphologically defined plant pathogenic fungi and oomycetes of the highest scientific and economic importance ([9,20]).

Species	DNA Target	Real-Time Format	LOD (Limit of Detection)	References
**Plant Pathogenic Fungi**				
*Magnaporthe oryzae*	18S-28S rDNA	SybrGreen	0.069 pg of genomic DNA extracted from fungal culture	[78]
*Botrytis cinerea*	IGS	TaqMan	0.02 pg of genomic DNA extracted from fungal culture and from infected plant material	[79]
	IGS	SybrGreen	6.3 pg of genomic DNA extracted from fungal culture	[80]
	*RPB2*	EvaGreen	1.55 pg from infected plant material	[81]
	*Cutinase A*	SybrGreen	0.2 pg genomic DNA extracted from fungal culture	[79,82]
*F. culmorum*	Mitochondrial *Cox2*	TaqMan	0.005−0.05 pg of genomic DNA extracted from fungal culture	[83]
*Fusarium graminearum*	*TEF-1α*	SybrGreen	0.1 pg genomic DNA extracted from fungal culture	[84]
	Anonymous	TaqMan	0.09 pg genomic DNA extracted from fungal culture	[85]
*F. oxysporum*	*TEF-1α*	SybrGreen	1 pg of genomic DNA extracted from fungal culture	[86]
*Zymoseptoria tritici (former Mycosphaerella graminicola)*	rDNA	SybrGreen	1 pg of DNA extracted from infected leaf samples	[87]
	microsatellite repeats	SybrGreen	0.01 pg of genomic DNA extracted from fungal culture0.05 pg of DNA extracted from infected leaf samples	[88]
*Colletotrichum* *acutatum*	ITS1 rDNA	TaqMan	0.05 pg of genomic DNA extracted from fungal culture and 12 pg per 100 mg plant material.	[89]
	ITS rDNA	SybrGreen	0.02 pg from infected plant samples	[90]
**Plant Pathogenic Oomycetes**				
*Phytophthora infestans*	ITS rDNA	TaqMan	0.1 pg extracted from pure cultures of *P. infestans*	[91]
	ITS rDNA	SybrGreen	0.5 pg/μL	[92]
*Hyaloperonospora arabidopsidis*	single-copy *Hpa* gene	SybrGreen	not determined	[93]
*Phytophthora ramorum*	ITS rDNA	SybrGreen	0.012 pg of genomic DNA extracted from pathogen biomass	[94]
	IGS between *Cox II* and *Cox I*	TaqMan	0.001 pg of genomic DNA extracted from pathogen biomass	[95]
	*Ypt1*	TaqMan (multiplex)	0.1 pg of genomic DNA extracted from pathogen biomass	[96]
*Phytophthora sojae*	ITS rDNA	SybrGreen	1 pg of genomic DNA extracted from pathogen biomass	[97]
	ITS rDNA	SybrGreen	0.001 pg/μL of genomic DNA extracted from pathogen biomass	[98]
*Phytophthora capsici*	ITS rDNA	SybrGreen	0.01 pg of genomic DNA extracted from pathogen biomass	[99]
*Plasmopara viticola*	ITS rDNA	TaqMan	0.1 pg of genomic DNA extracted from pathogen biomass	[100]
*Phytophthora cinnamomi*	*LPV*	SybrGreen (nested PCR)	0.02 pg of genomic DNA extracted from pure cultures of *P. cinnamomi*	[101]
*Pythium ultimum*	ITS rDNA	SybrGreen	0.005 pg from contaminated soil	[102]
	ITS rDNA	SybrGreen	Not determined	[103]
*Pythium ultimum var. ultimum*	ITS rDNA	SybrGreen	0.013 pg μL^−1^ from infected plant tissue	[104]

**Table 2:** Examples of qPCR approaches for cryptic species, formae speciales, and anastomosis groups of plant pathogenic fungi.

Species	DNA Target	Real-Time Format	LOD (Limit of Detection)	References
**Cryptic Species**				
*F. graminearum* s.s.	*MAT*	TaqMan	0.64 pg of genomic DNA extracted from fungal culture	[105]
	Mitochondrial *Cob*	TaqMan	0.2–0.06 pg of genomic DNA extracted from fungal culture	[53]
*Colletotrichum kahawae*	*GAPDH*	TaqMan	0.08 pg μL^–1^ of genomic DNA extracted from fungal culture	[106]
**Formae Speciales**				
*F. oxysporum f. sp. lycopersici*	*SIX1*	TaqMan	0.44 pg of genomic DNA extracted from fungal culture	[107]
*F. oxysporum* *f. sp. cubense race 4*	Anonymous	SybrGreen	0.1 pg of genomic DNA extracted from fungal culture	[108]
	Putative virulence gene	TaqMan	24 plasmid copies of target DNA per reaction tube	[109]
*F. oxysporum* *f. sp. phaseoli*	virulence factor *ftf1*	TaqMan	2 pg of genomic DNA extracted from fungal culture	[110]
*F. oxysporum* *f. sp. spinaciae*	IGS	TaqMan	0.01 pg of genomic DNA extracted from fungal culture	[111]
**Anastomosis Groups**				
*Rhizoctonia solani* AG-1 IA	ITS rDNA	SybrGreen	1 pg of genomic DNA extracted from fungal cultures	[112]
*Rhizoctonia solani* AG-3	ITS rDNA	TaqMan	0.006–0.009 pg DNA *µ*L^−1^ in naturally contaminated soil	[76]
